# Lateral Controlled Doping and Defect Engineering of Graphene by Ultra-Low-Energy Ion Implantation

**DOI:** 10.3390/nano13040658

**Published:** 2023-02-08

**Authors:** Felix Junge, Manuel Auge, Zviadi Zarkua, Hans Hofsäss

**Affiliations:** 1 II. Institute of Physics, Georg-August-Universität Göttingen, 37077 Göttingen, Germany; 2 Quantum Solid State Physics, KU Leuven, 3001 Leuven, Belgium

**Keywords:** graphene, ion implantation, ion implantation simulations, 2D materials, IMINTDYN

## Abstract

In this paper, the effectiveness of ultra-low-energy ion implantation as a means of defect engineering in graphene was explored through the measurement of Scanning Kelvin Probe Microscopy (SKPM) and Raman spectroscopy, with boron (B) and helium (He) ions being implanted into monolayer graphene samples. We used electrostatic masks to create a doped and non-doped region in one single implantation step. For verification we measured the surface potential profile along the sample and proved the feasibility of lateral controllable doping. In another experiment, a voltage gradient was applied across the graphene layer in order to implant helium at different energies and thus perform an ion-energy-dependent investigation of the implantation damage of the graphene. For this purpose Raman measurements were performed, which show the different damage due to the various ion energies. Finally, ion implantation simulations were conducted to evaluate damage formation.

## 1. Introduction

As revolutionary materials with unique mechanical, thermal, and electrical properties, graphene and other 2D materials such as transition-metal dichalcogenides (TMDs) have been the subject of extensive research and development in various fields, from electronics to biomedicine. Some of the new applications include for example sensors for biomolecules and environmental contaminants [1,2,3,4], spintronic devices [5] or water cleaning [6]. To further modify the electrical, magnetic, structural or optical properties of 2D materials, foreign atoms can be introduced into the crystal lattice [7,8,9]. This can be achieved using various methods, such as adapting the growth process or diffusing atoms into the material [10,11]. A direct approach frequently used in the semiconductor industry is ion implantation. In this process, foreign atoms can be introduced into the materials in a targeted manner without altering the growth process, or reliance on chemistry or diffusion, which ensures a very atom specific incorporation. Conventional ion implantation (>1 keV), however, are insufficient for implanting in 2D materials, due to required implantation energies of only a few 10 eV [12]. Since the generation of an ion beam with such low energy and reasonable implantation currents (<10 nA on the sample) is a great challenge, the deceleration of the ions in front of the sample by applying an electrical potential to the sample a promising approach to reach the ultra-low energy regime (few 10 eV). This has the advantage that, in contrast to deceleration by a capping layer [13], no recoil atoms from the capping layer enter the sample and thus only the desired atomic species is implanted and no contamination occur. For graphene and TMDs it has already been shown that properties and damage of the sample can be successfully tuned by means of laterally uniform ULE ion implantation [14,15,16,17,18,19,20]. To further expand the capabilities of ULE ion implantation, we demonstrate how the surface of a graphene sample can be selectively modified by ion beams using laterally controlled implantation [21], paving the way for more complex implantation structures and thus the possibility of fabricating electrical 2D devices and the exploration of new scientific questions. For 2D target materials, electrostatic deflection has significant advantages compared to a conventional shadow mask. Indeed, when using physical masks, recoils can occur, which are also incorporated into the graphene. Another disadvantage of a shadow mask are the electrical field inhomogeneities, which occur at the edges of the mask due to the high electrostatic potential gradient required for the ion deceleration. Due to these inhomogeneities, sharp transition regions are prevented. This is amplified because a physical mask should also hover over the samples to avoid damage to the graphene. Electrostatic masking allows a contactless shielding of the sample and provides therefore a good possibility for the lateral selective implantation of one sample. Another application is the generation of an electrical potential gradient along the sample to obtain different implantation energies at different sample positions. This creates an opportunity to study the effects of implantations, such as damage formation, along a continuous ion energy gradient while all other experimental conditions, such as sample preparation, are the same. In this paper the electrostatic masks were used for boron (B) implantations of graphene to generate a transition between pristine and implanted regions on graphene. Additionally helium (He) was implanted in another graphene sample with an applied potential gradient to study the effects of different implantation energies.

Simulations of He implantation in graphene were conducted to provide a better understanding of the processes involved. Here, the damage formation can also be investigated depending on the implantation energy and the used fluence.

## 2. Materials and Methods

### 2.1. ULE Ion Implantation

The ion implanter ADONIS [22,23] was used for the implantations, which is currently the only accelerator in the world that can provide these ultra-low energies and can be used for direct implantation into 2D materials. In this setup, the ions are first accelerated with 30 kV, mass selected with the help of a 90°-sector magnet, with a mass resolution of M/ΔM=150 and finally slowed down again to achieve implantation energies of several 10 eV. This deceleration is carried out by placing the sample at the voltage of the ion source anode, minus the desired implantation voltage [24]. Thus, it can be ensured that the ions impinge the sample with a maximum energy, which is set beforehand in the range of 10 eV–600 eV. To exclude different energies due to fluctuations in different power supplies, the same power supply is used for acceleration and deceleration. Due to the high mass resolution only the desired ion species is implanted. Neutral particles are removed from the beam before entering the implantation chamber, so that all particles can be decelerated in front of the sample. All implantations were carried out at room temperature in ultra-high vacuum (<10^−8^ mbar).

### 2.2. Introducing Lateral Inhomogeneities

#### 2.2.1. Electrostatic Masking

To achieve electrostatic masking, the deceleration unit described in [21] is used. Here, an electrode is hovering at a distance of about 1 mm above the sample and is set to +100 V or +300 V with respect to the sample potential. This deflects the incoming ions, which have an energy of a few 10 eV at this point, and pushes them away from one side of the sample. A typical simulation for 100 V can be found in [21]. The sample itself is at −20 V in respect to the 30 kV accelerating voltage, which allows the ions to be implanted with a maximum energy of 20 eV.

Monolayer graphene on SiO_2_ and Ni were used as samples, which were obtained from Graphene Supermarket (Graphene Laboratories Inc., Ronkonkoma, NY, USA, https://www.graphene-supermarket.com). B was implanted with fluences of $5\times 10^{14}$ atcm2 and $1\times 10^{15}$ atcm2. The samples were then analyzed by Scanning Kelvin Probe Microscopy (SKPM) measurements to determine the surface potential along the sample to analyze the sharpness of the transition between the implanted and un-implanted region.

#### 2.2.2. Potential Gradient

To create an energy gradient for studying the damage of graphene when implanted with different energies, 20 nm copper contacts were first applied by sputter deposition on both sides of the graphene. Then, a constant voltage source was used to apply a potential difference of 100 V. The ground contact on the graphene was set to 0 V with respect to the 30 kV anode of the source, so that the incoming ions have an energy between 100 eV–0 eV. The current flow through the graphene layer results in a linear potential gradient along the surface. As sample, monolayer graphene on SiO_2_ was used, so that the SiO_2_ layer acts as an insulator and the voltage drops only through the graphene layer. The samples were also obtained from Graphene Supermarket. He ions were used for the implantation with a fluence of 1×1015 atcm2. The feasibility of laterally uniform He implantation into graphene has been shown in previous publications [25]. 

### 2.3. Sample Charaterization

For the measurement of the surface potential (SKPM measurements) of the sample after implantation using the electro static mask, measurements were performed with an Atomic Force Microscope, type MFP-3D Origin+ from Oxford Instruments Asylum Research (Oxford Instruments GmbH, Wiesbaden, Germany), in Scanning Kelvin Probe Microscopy (SKPM) non-contact constant height mode. Here the cantilever is held at a constant height above the specimen and the force on the cantilever is measured based on an applied voltage between the cantilever and the specimen. Subsequently, the value for each measurement was averaged over an area of 5 µm × 5 µm. Normal AFM measurements were not recorded separately and the selected resolution was sufficient for averaging of the surface potential but not for specific height analyses. The damage formation was determined using Raman spectrometry. Raman spectra were measured using a confocal Raman microscope (Monovista CRS+, S&I GmbH, Warstein, Germany) equipped with a 532 nm Nd:YAG laser. The laser was directed onto the sample surface through an objective (OLYMPUS, X43 100×, N.A. 0.7), with the maximum laser power remaining below 1 mW in order to avoid laser-induced modification. All the measurements were obtained in ambient conditions, at room temperatur.

## 3. Results and Discussion

### 3.1. Change of Surface Potential by B Implantation

**Figure 1:**
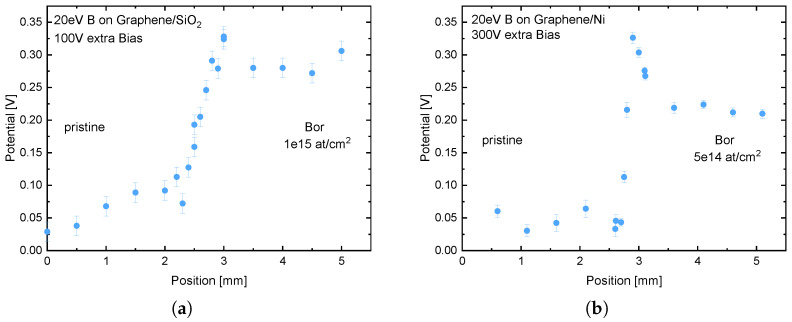
(**a**) SKPM measurement of a graphene layer on SiO_2_ previously implanted with 1×1015 atcm2 with B with an energy of 20 eV. In addition, only a part of the sample was implanted using an electrostatic mask. The voltage at the mask was set to +100 V corresponding to the sample bias. (**b**) SKPM measurement of a graphene layer on Nickel previously implanted with $5\times 10^{14}$ atcm2 with Boron at an energy of 20 eV. The voltage for masking was set to +300 V in respect to the sample bias. The comparison shows a sharper transition region (≈1 mm to ≈0.5 mm) with increasing deflection voltage. The position indicates the relative position to the edge of the sample.

The SKPM measurements, shown in Figure 1, reveal a change in the surface potential between the pristine and implanted region of the sample. It can be observed that the level of surface potential change correlates with the fluence used. Due to the stronger deflection of the ions at the 300 V mask (Figure 1b), a significant reduction of the transition zone from about 1 mm to below 500 µm has been achieved, compared to the 100 V mask. In addition, an increase in the surface potential, and thus the doping, at the interface can be seen at higher deflection voltages. This is due to the fact that the ions, which are deflected, are pushed to the sides and generate a higher fluence there. This only occurs on the implanted side, since the other side is completely shielded by the hovering electrode. An exact simulation with SIMION [26] of the ion trajectories can be found in [21]. Based on the magnitude of the surface potential, the high fluence of the region next to the transition can also be determined, since this potential corresponds to the surface potential of the $1\times 10^{15}$ atcm2 implanted sample. Thus, in the case of the 300 V deflection, the fluence directly at the interface is about twice as high as expected. This can also be used to dope various doping levels in different areas on the sample. By using different substrates (SiO_2_ and Ni), and observing a similar change of the surface potential, both in values and in general behavior, it can be concluded that this effect is due to the doping of the graphene and not to the effects of the substrate.

### 3.2. Energy Dependent Defect Formation by He Implantations

**Figure 2:**
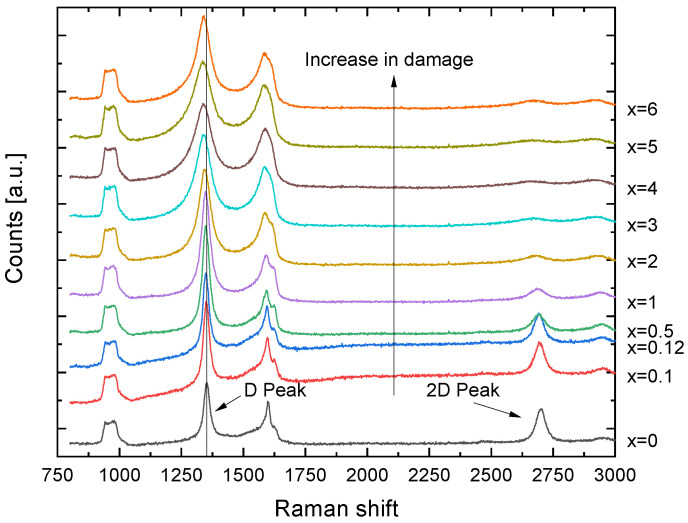
Raman spectra of the Helium implanted sample. The spectrum was measured on different positions on the sample. Here, x is in mm, where x=0 describes the position directly at the 0 V contact. With higher x also the He energy increases from $E_{He}\approx 0$ eV at x=0 mm to $E_{He}\approx 100$ eV at x > 6 mm. A clear shift of the D-peak and a broadening due to a higher implantation energy at larger x can be seen.

In Figure 2 different Raman spectra for different sample surface positions of the He implanted sample are shown. The positioning describes the distance from the one copper electrode connected to the 0 V. Therefore, lower positions corresponds to lower implantation energy. The gradient can be approximately assumed to be linear, since the resistance across the graphene layer should not change. Along the sample surface, a clear shift of the D-peak as well as a broadening can be seen. Another indication for a large damage is provided by the 2D-peak, which gets smaller with higher He energy until it almost disappears. For x > 3 mm the graphene becomes more and more amorphous, presumably due to the high fluence of 1×1015atcm2. This is shown by the broadening of the G-peak and the merging with the D’-peak due to the broadening of the D band, which indicates the loss of the crystal structure is thus also a sign of higher damage to the graphene [27,28]. A typical Raman spectrum of graphene from Graphene Supermarket before and after an ion implantation can be found in [29]. Similar to our results, a decrease in the 2D-peak and an increase of the D-peak after the implantation was observed as damage increases. It should be noted that the first measuring point already shows a clear damage formation, since the 2D-peak is already smaller than the D-peak although at this position the implantation energy of around 1–5 eV is too small for vacancy formation. However, intercalation of He between the graphene and the substrate can still occur which leads to damage. Moreover, the graphene was probably slightly damaged by the sputtering process when applying the copper contacts. These measurements only serve as a proof of concept, since due to the strong initial damage caused by the copper contacts and the high fluence of the implanted ions, it is not possible to make a quantitative statement about the damage compared to the implantation energy. This is partly because the exact field gradient over the sample was assumed to be linear, but can deviate from this due to various disturbances in the graphene. Nevertheless, these measurements show a clear trend towards more damage at higher ion energies, similar to results of laterally uniform implantations [20].

### 3.3. Simulations

To obtain a more profound understanding of the processes during implantation, simulations were performed using IMINTDYN [30], a binary collision approximation (BCA) Monte Carlo program for simulation of ion solid interactions based on SDTrimSP [31]. As most BCA simulation codes IMINTDYN also assumes an amorphous target structure. The interaction potential is the screened Kr-C potential and for electronic stopping the SRIM2013 database is used. IMINTDYN as well as SDTrimSP can perform dynamic simulations, which take stoichiometry changes, erosion and deposition during ion irradiation into account. The quality of different BCA simulation codes at lower ion energies in comparison with experimental data has been investigated by one of the authors in the past [32]. SDTrimSP and IMINTDYN simulations have been successfully applied to model surface pattern formation by low energy ion irradiation at grazing ion incidence [33,34]. We have also used BCA Monte Carlo simulations previously to model ultra low energy ion irradiation of graphen and other 2D materials to estimate the erosion effects and the ion retention as function of ion energy [29]. A special new feature of IMINTDYN is the possibility to insert vacancies as a target atom species. The vacancy does not influence the path direction of a moving particle but just increases its mean free path length. This offers the advantage that vacancies generated by recoil collisions and vacancies annihilated by stopped atoms dynamically change the composition of the sample and thus provides a more accurate representation of the final layer structure with increasing ion fluence. Furthermore, vacancies allow to model 2D materials much more realistic when the space in the center of six-ring atoms arrangements and the space between 2D layers is filled with vacancies. In other BCA simulations 2D materials are represented simply as homogeneous amorphous layers. The graphene monolayer with layer spacing of 3.35 Å, is simulated as a 1.1 Å thick carbon layer, followed by two layers of vacancies each 1.1 Å thick. The deeper layers, and thus the substrate, consist of SiO_2_. The sublimation energy of 7.428 eV is used as the bulk binding energy (or surface binding energy) of carbon. This also corresponds to the vacancy formation energy of graphene [35]. For Si, O and other atoms we use the corresponding sublimation energies.

Since IMINITDYN does not simulate a lattice structure, but assumes the layers as amorphous, the graphene layer was adjusted slightly to take the hexagonal arrangement of the atoms into account. Because of the lattice structure there is the possibility that an ion flies through the middle of the carbon ring without colliding with an atom. Therefore, the first layer in the simulation consists of 2/3 C-atoms and 1/3 vacancies since two C-atoms and one empty center are to be assigned to one carbon ring. The density of the layer, with a layer spacing of 3.35 Å, was then increased to 0.4965 atÅ3, so that the atomic density of carbon atoms again corresponds to graphene. In the interpretation of the results, however, it must be taken into account that the first layer consists of only 66.7% carbon followed by two layers consisting solely of vacancies. In addition, not only collisions with the atoms that can hit directly but also collisions with nearest neighbor and next-nearest neighbor atoms (so-called weak collisions) were taken into account.

**Figure 3:**
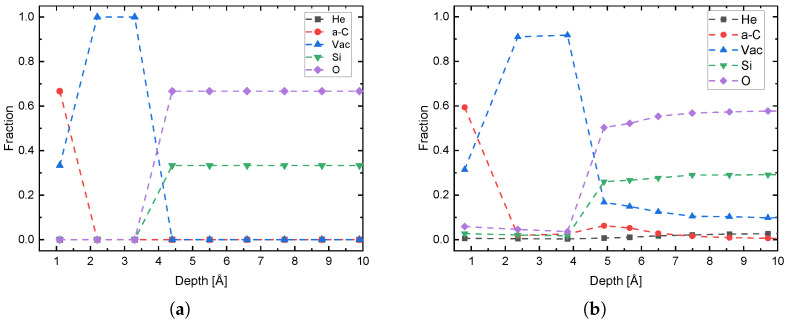
Start condition of the IMINTDYN simulation of graphene on SiO_2_ (**a**) and after He implantation at 100eV with a fluence of $1\times 10^{15}$ atcm2 (**b**). After the implantation, damage to the graphene due to He, vacancies and substrate atoms is clearly visible in the top atomic layer.

**Figure 4:**
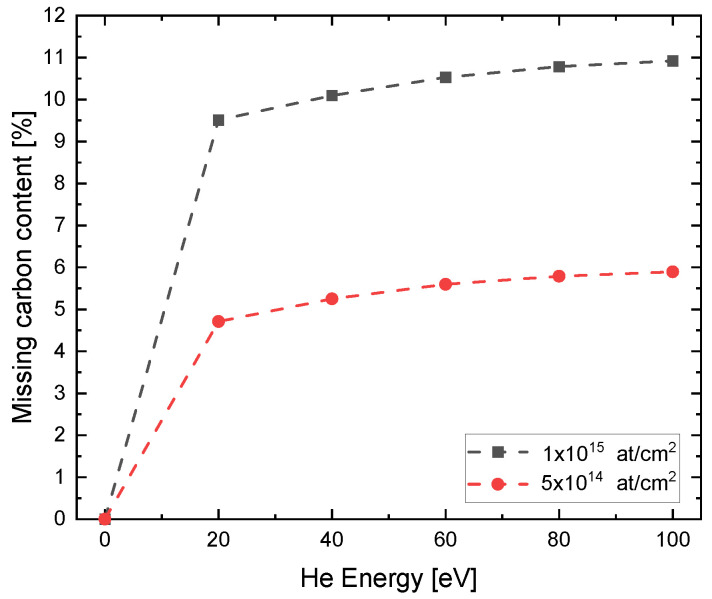
Values of missing carbon content in the top layer versus He implantation energy from the IMINTDYN simulations. A clear trend towards higher damage to the graphene at higher He energy is becoming apparent.

The He ion energies 20, 40, 60, 80 and 100 eV were simulated with fluences of both $5\times 10^{14}$ atcm2 and $1\times 10^{15}$ atcm2. The results in the composition of the first nanometer of the sample are shown exemplary in Figure 3. It is shown that the carbon content in the first layer decreases after implantation and is replaced by either vacancies, He or atoms from the substrate. In this extreme case, the carbon concentration drops by 10.9% from 66.7% to 59.4%. In addition, it can be observed that some of the carbon atoms can be incorporated into the substrate by recoil formation and that the uppermost layers of the substrate are also damaged, seen in a increase in vacancies and loss of Si and O. This shows that already at these low energies a certain amount of recoils are generated which are incorporated into the underlying layers, and therefore the application of a capping layer to slow down the ions can lead to further damage of 2D materials and to incorparation of unwanted foreign atoms. The trend of the carbon decrease with higher energy is shown in Figure 4. It can be noticed that with higher He energy and higher fluence, more carbon is removed from the first layer. The missing carbon content in the first layer in relation to the undamaged graphene can be comprehended as a measure of damage. Here the simulation confirms the higher defect density at higher implantation energies. In addition, it can be seen that in this energy regime, the fluence has a significantly greater influence on damage formation. Therefore, it is not surprising that the Raman spectrum shifts toward amorphous carbon for the fluence used in the experiment. The damage is caused on the one hand by the creation of vacancies and on the other hand by the incorporation of free atoms, either He or atoms of the substrate, which can be incorporated into the uppermost layers due to sputtering processes.

**Figure 5:**
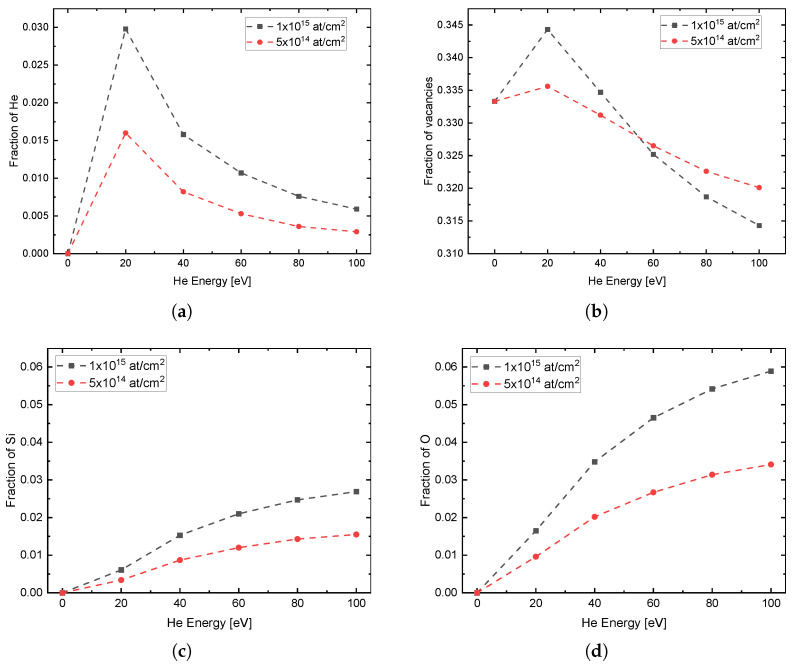
Concentrations of impurity atoms (**a**) He, (**b**) vacancies, (**c**) Si, and (**d**) O in the top graphene layer after implantation of He with different energies and fluences of $1\times 10^{15}$ atcm2 and $5\times 10^{14}$ atcm2.

Figure 5 shows the fractions of introduced He, vacancies, Si, and O from the substrate into the top graphene layer after implantations versus implantation energy. For He (Figure 5a) it can be seen that a maximum is reached at 20 eV. This is due to the fact that at higher energies the He is implanted deeper into the sample and thus penetrates the graphene layer more and more. For the vacancies (Figure 5b) it can be seen that the vacancies first increase from the initial value of 33.3%. Subsequently, the concentration falls again, with higher decrease with higher fluence, so that initially at the fluence of $1\times 10^{15}$ atcm2 and energies of up to just below 60 eV more vacancies are generated. At higher energies, more vacancies were generated in the top layer for the lower fluence of $5\times 10^{14}$ atcm2. For Si (Figure 5c) and O (Figure 5d) it can be seen that with increasing energy the fraction of these substrate atoms in the graphene layer also increases, also more pronounced with higher fluence. The decrease in the vacancy concentration with a simultaneous decrease in carbon can be explained by the introduction of Si and O into the graphene layer, with O having a significantly greater effect. This effect is more pronounced at higher energies and can be explained by the scattering kinematics. When He hits Si or O, the resulting recoil can only go in the forward direction. In order to be incorporated into the graphene, a further scattering of the generated recoil with a substrate atom must subsequently take place. To have enough energy for the second scattering in the cascade, the first collision of He with Si or O must transfer sufficient high energy, thus this process becomes more likely with higher He energies. Another process is the backscattering of He in deeper substrate layers and the subsequent collision of He with Si or O on its way back. In this way, Si or O is also scattered towards the surface and can be incorporated there. The proportion to which these processes take place depends on the He energy. Thus, the process via the cascade has a fraction of 83% (17% via a direct collision with backscattered He) at 100 eV and of 53% (47% via a direct collision with backscattered He) at 20 eV. The energy transfer to C, O and Si can be calculated by the kinematic factor and has its maximum at 180° backscattering angle. Under these conditions, the maximum transferred energy is 75% for C, 64% for O and 43.75% for Si. Due to the 1/E^2^ dependency of the backscattering cross section, where E is the projectile energy, the high backscattering yield is reasonable in the ultra-low energy regime. The fact that more O than Si is incorporated into the graphene is due to the double O concentration in the substrate (SiO_2_) as well as to the lower mass of O. Therefore the backscattering in the second collision of the cascade is more probable.

## 4. Conclusions

We have shown that, by using electrostatic masks and potential differences, graphene can be doped with different fluences in only one implantation step. In addition, by adjusting the deflection voltage, the transition region between the doped and un-doped regions can be set. This is a great step towards the production of electronical graphene devices such as transistors using ion implantation. By being able to implant a sample at different energies at different surface positions, future measurements and investigations can be accelerated and eliminates unintended experimental variations in sample preparation as only one sample needs to be implanted instead of multiple.

These methods can be transferred to other 2D materials such as TMDs and therefore can be helpful for a better investigation and manipulation of these materials. Further experiments to sharpen the transition zone are planned. The simulations carried out can confirm the findings from the experiments and are therefore a valuable tool for predicting the processes during ultra-low energy ion implantation of 2D materials.
